# New WGS data and annotation of the heterosomal *vs*. autosomal localization of *Ostrinia scapulalis* (Lepidoptera, Crambidae) nuclear genomic scaffolds

**DOI:** 10.1016/j.dib.2018.08.011

**Published:** 2018-08-09

**Authors:** Louise Brousseau, Sabine Nidelet, Réjane Streiff

**Affiliations:** a CBGP, INRA, CIRAD, IRD, Montpellier SupAgro, Univ Montpellier, Montpellier, France; b IRD, UMR DIADE Diversité - Adaptation - Développement, 911 Avenue Agropolis, BP64501, 34394 Montpellier, France

**Keywords:** *Ostrinia scapulalis*, Genome, NGS, HiSeq2500, Depth analysis, AD-ratio, Structural annotation, Sex-chromosome, Z-heterosome, Autosomes

## Abstract

Here, we introduce new whole-genome shotgun sequencing and annotation data describing the autosomal *vs*. Z-heterosomal localization of nuclear genomic scaffolds of the moth species *Ostrinia scapulalis*. Four WGS libraries (corresponding to 2 males and 2 females) were sequenced with an Illumina HiSeq2500 sequencing technology, and the so-called *‘AD-ratio’* method was applied to distinguish between autosomal and Z-heterosomal scaffolds based on sequencing depth comparisons between homogametic (male) and heterogametic (female) libraries. A total of 25,760 scaffolds (corresponding to 341.69 Mb) were labelled as autosomal and 1273 scaffolds (15.29 Mb) were labelled as Z-heterosomal, totaling about 357 Mb. Besides, 4874 scaffolds (29.07 Mb) remain ambiguous because of a lack of *AD-ratio* reproducibility between the two replicates. The annotation method was evaluated *a posteriori*, by comparing depth-based annotation with the exact localization of known genes. Raw genomic data have been deposited and made accessible via the EMBL ENA BioProject id PRJEB26557. Comprehensive annotation is made accessible *via* the LepidoDB database (http://bipaa.genouest.org/sp/ostrinia_scapulalis/download/genome/v1.2/).

**Specifications Table**

**Table:** 

Subject area	Biology
More specific subject area	Genomics, Bioinformatics
Type of data	DNA sequence reads and annotation table
How data was acquired	Shotgun whole genome sequencing (Illumina HiSeq2500)
Data format	Raw (2 × 125 raw reads) and Analyzed (Scaffolds annotation)
Experimental factors	Wild-type specimens collected in the field
Experimental features	Genome: DNA sequencing
Data source location	Abbeville, France (50° 8′11.03′′N; 1°49′49.22′′E)
Data accessibility	Raw reads are accessible through the EMBL ENA BioProject id PRJEB26557 (https://www.ebi.ac.uk/ena/data/view/PRJEB26557)
	Annotation data are made accessible *via* the LepidoDB database (http://bipaa.genouest.org/sp/ostrinia_scapulalis/download/genome/v1.2/)
Related article	B. Gschloessl, F. Dorkeld, P. Audiot, A. Bretaudeau, C. Kerdelhué, R. Streiff (2018) *De novo* genome and transcriptome resources of the Adzuki bean borer *Ostrinia scapulalis* (Lepidoptera: Crambidae). *Data in Brief*. doi 10.1016/j.dib.2018.01.073[1]

**Value of the data**

● This article enriches and updates the annotation of *Ostrinia scapulalis* (Lepidoptera) nuclear genome recently published by Gschloessl et al. [1] with an accurate annotation of the chromosomal localization of the scaffolds constituting the nuclear genome assembly.
● The new genomic data acquired here (whole-genome shotgun sequencing of four libraries, two males and two females) will enrich the public sequence database for this species.
● WGS sequencing depth analysis is a promising method to retrieve the autosomal or heterosomal localization of assembly fragments (scaffolds or contigs) obtained through *de novo* assembly.
● The annotation data released here provide key information about the autosomal *vs.* Z-heterosomal localization of scaffolds described in Gschloessl et al. [1].
● Such annotation is of great value for future evolutionary studies, as genome-wide population genomics analyses (e.g. continent-scale phylogeography, host-plant adaption studies etc.) may be dramatically sensitive to the confounding influence of autosomal and heterosomal evolutionary histories (because of different inheritance, ploïdy levels, recombination rates, effective population size and genetic drift).

## 1 Data

The dataset described here is composed of new whole-genome sequencing (WGS) data (paired-end sequencing of four libraries with an Illumina HiSeq2500 sequencing technology) and a new annotation of autosomal and heterosomal scaffolds of the nuclear genome of the moth species *Ostrinia scapulalis.* These new data are complementary to the scaffold-level nuclear genome (hereafter *OSCA*) recently assembled for this species [1]. Specifically, we applied the *AD-ratio* method originally developed by Bidon et al. [2] to compare sequencing depth between male and female libraries, and we introduce an accurate labelling (autosomal *vs*. Z*-*heterosomal) of the scaffolds of *OSCA* genomic reference which enriches preliminary structural and functional annotations described in Gschloessl et al. [1].

## 2 Experimental design, materials, and methods

### 2.1 Species model

*O. scapulalis* (i.e. the Adzuki bean borer) is a phytophagous moth species living on a variety of dicotyledon plants (e.g. *Humulus lupulus*, *Artemisia vulgaris*, *Cannabis sativa*) across Europe, and phylogenetically close to the European corn borer (*O. nubilalis)*, a major maize pest worldwide. In this species, 31 pairs of chromosomes are expected (30 autosomal pairs and one heterosomal pair) with a ZZ/ZW sex determination: males are homogametic (ZZ) and females are heterogametic (ZW).

### 2.2 Sampling and DNA extraction

*O. scapulalis* diapausing larvae were collected in stems of wild mugwort in northern France (Abbeville, Picardie) and stored in 95% ethanol at − 20 °C. Genomic DNA (gDNA) was extracted using BioBasic ‘96-well plate animal genomic DNA mini-preps’ extraction kits (Euromedex) according to manufacturer׳s instructions. gDNAs were quantified using a NanoDrop 8000 Spectrophotometer (Thermo Scientific). The sex of each sample was characterized according to the molecular method described in Orsucci et al. [3]: sex-linked microsatellite markers, ONW1 and ONZ1 (specific of W and Z heterochromosomes respectively), were amplified simultaneously using the Multiplex PCR Master Mix (Qiagen). Four specimens were finally retained among the best quality DNAs: two males (IDs 12098 and 12114) and two females (IDs 12099 and 12111).

### 2.3 Library preparation and sequencing

Four libraries (one per sample) were prepared according to the ‘TruSeq Nano DNA Library Preparation Guide’ (Illumina, https://support.illumina.com/downloads/truseq-nano-dna-library-prep-guide-15041110.html), starting with 100 ng of gDNA per library. According to manufacturer׳s instruction, library preparation workflow included: (1) gDNA fragmentation with a Covaris S220, (2) libraries end-repair and sizing, (3) 3′ ends adenylation, (4) adapters ligation, (4) DNA fragments enrichment, and (5) libraries normalization and pooling. At the end of the enrichment step (4), libraries quality and quantity were evaluated using both a ‘DNA 1000 ship’ (‘Agilent Technologies 2100 Bioanalyzer’) and a ‘KAPA Library Quantification Kit’ (KAPA Biosystems). Libraries varied between 515 and 533 bp in size, and were pooled 2 by 2 (one male and one female library per pool) in 20 nM equimolar mixture. Shotgun libraries were sequenced with an Illumina HiSeq2500 paired-end (2×125 bp) sequencing technology by the ISO9001:2008 Montpellier Genomix facility (MGX, France, http://www.mgx.cnrs.fr).

### 2.4 Bioinformatics pipeline

The bioinformatics pipeline is detailed in Supplementary file 1. In brief, raw reads were cleaned and mapped against the reference nuclear genome *OSCA* as follow:

(1) Reads that did not pass *Illumina* chastity filter (i.e. purity filter PF) were discarded with *zcat* and *grep*.
(2) phiX control reads were removed by mapping raw reads against phiX genome with bowtie2 [4]: only unmapped reads were used in the following.
(3) Individual bases of low quality (*phred-score* <25) were masked using fastq_masker (http://hannonlab.cshl.edu/fastx_toolkit/) with parameters − q 25 − Q 33.
(4) Reads of low quality and orphan reads were discarded using sickle [5], with parameter − q 25.
(5) Cleaned reads were mapped against the reference nuclear genome *OSCA* [1], separately for each library, using bwa aln and bwa sampe [6].
(6) SAM files (.sam) were converted into sorted BAM with samtools view (with parameter − q 20 to discard multiple mapped reads) and samtools sort [7].
(7) Multiple BAM (.mpileup) were generated with samtools mpileup and converted into tabular ‘synchronized’ files with ‘mpileup2sync’ perl script originally implemented in Popoolation2 [8].

Synchronized files were further handled with perl and R to estimate base depth, per-scaffold mean depth, and to compute *AD-ratios* between homogametic and heterogametic libraries, see Supplementary file 2. The *AD-ratio* method [2] is conceptually based on the simple assumption that the ratio of sequencing depth between homogametic (here, male ZZ) and heterogametic (here, female ZW) libraries - standardized by the number of mapped reads for each library - would be 1 for autosomal scaffolds, 2 for Z-heterosomal scaffolds and 0 for W-heterosomal scaffolds, (see Supplementary file 3 for additional information about scaffold-specific AD-ratio estimation). Note here that the W-chromosome is absent from the *OSCA* nuclear reference which was drawn from a single male (ZZ) [1].

**Fig. 1:**
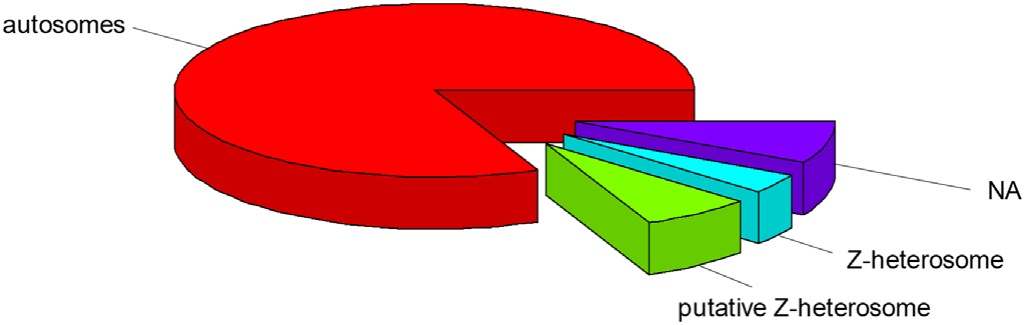
Total assembly length (~ 420 Mb) partitioning into autosomal (~ 342 Mb), Z-heterosomal (~ 15 Mb), putatively Z-heterosomal (~ 29 Mb) and un-annotated (~ 33 Mb) genomic regions.

A total of 25,760 scaffolds (corresponding to 341.69 Mb) were identified as autosomal and 1273 scaffolds (15.29 Mb) were identified as Z-heterosomal, totaling about 357 Mb, Fig. 1. Besides, 4874 scaffolds (29.07 Mb) were ambiguously annotated because of a lack of AD-ratio reproducibility between the two replicates, putting thus the emphasis on the necessity to use two independent biological replicates. Last, 18,831 scaffolds remained un-annotated, because of insufficient mapping depth (< 4*X* in average) in one or several libraries. They represent ~ 33 Mb, which corresponds to ~ 8% of total assembly length, indicating that the subset of un-annotated scaffold is largely enriched in short scaffolds. Comprehensive annotation is provided in Supplementary file 4 and is made publically-available *via* the LepidoDB database (http://bipaa.genouest.org/sp/ostrinia_scapulalis/download/genome/v1.2/).

### 2.5 A posteriori validation

We compared the ‘blinded’ annotation based on *AD-ratios* for scaffolds holding either autosomal or Z-heterosomal known candidate genes, including six olfactory receptors, *OR1* to *OR6* [9], [10], and two Z-linked genes, *TPi* and *Kettin* respectively [11]. To do that, candidate genes were localized by blastn against the nuclear reference *OSCA* using the program blastall (*e*-value <10^−20^). The Z *vs.* autosomal annotation based on *AD-ratios* was totally consistent with the actual Z or autosomal localization of the candidate genes, even in cases of a lack of reproducibility between replicates (Supplementary file 5).
