# Supplementary material for: New WGS data and annotation of the heterosomal vs. autosomal localization of Ostrinia scapulalis (Lepidoptera, Crambidae) nuclear genomic scaffolds
Source: Data Brief. 2018 Aug 9;20:644–8. doi: 10.1016/j.dib.2018.08.011 (PMC6127984; doi:10.1016/j.dib.2018.08.011)
Supplement: Supplementary file 3 — Supplementary material [file mmc3.docx]

**Supplementary file 2. Mapping results**

*OSCA* nuclear genome is composed of 50 738 scaffolds, for a total assembly length of about 420 Mb. Here, between 69 802 497 (Lib 12111) and 70 941 222 (12114) reads per library were successfully mapped. Average depth per scaffold varied between 17.97 X ±50.34 (Lib 12098) and 18.14 X ±48.68 (Lib 12099), with low variability between libraries.

**
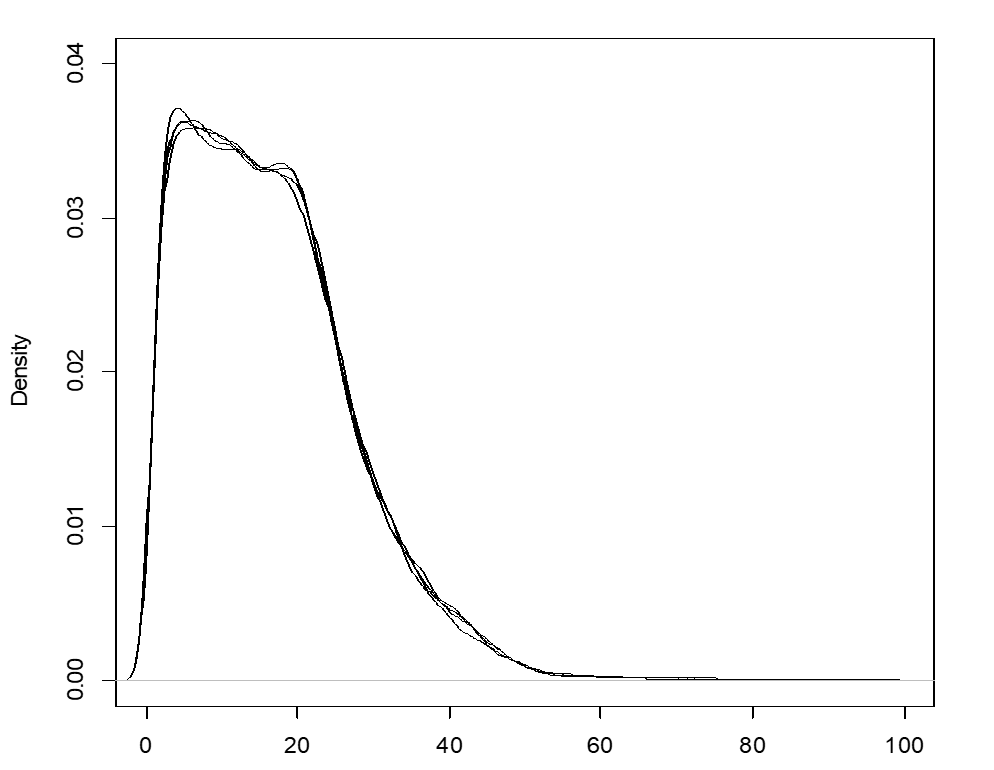
**

**Fig.** Distribution of average depth per scaffold for the different libraries Lib 12098, Lib 12099, Lib 12111 and Lib 12114.
