# Supplementary material for: New WGS data and annotation of the heterosomal vs. autosomal localization of Ostrinia scapulalis (Lepidoptera, Crambidae) nuclear genomic scaffolds
Source: Data Brief. 2018 Aug 9;20:644–8. doi: 10.1016/j.dib.2018.08.011 (PMC6127984; doi:10.1016/j.dib.2018.08.011)
Supplement: Supplementary file 4 — Supplementary material [file mmc4.docx]

**Supplementary file 3. AD-ratio Method and decision criteria**

For each scaffold, $AD-ratio=\frac{\frac{{AD}_{male}}{{Nr}_{male}}}{\frac{{AD}_{female}}{{Nr}_{female}}}$, where *AD_male_* and *AD_female_* are the scaffold’s average depth (AD) in male and female libraries respectively, and *Nr_male_* and *Nr_female_* the total number of reads mapped in the corresponding library. Because the four libraries were sequenced on two lanes, with one male and one female library per lane, we used the two lanes as two independent replicates of the *AD-ratio* for each scaffold. Scaffolds mapped only marginally in one or several libraries (average depth below 4X) were discarded. The remaining scaffolds were annotated according to the following criteria.

**Table: Annotation decision criteria, based on AD-ratio range in each replicate.**

| *AD-ratio* replicate1 | *AD-ratio* replicate2 | **Annotation** |
| --- | --- | --- |
| ]0-1.5[ | ]0-1.5[ | **-> Autosome** |
| ]0-1.5[ | [1.5-2.5[ | **-> Putative Z-heterosome** |
| [1.5-2.5[ | ]0-1.5[ | **-> Putative Z-heterosome** |
| [1.5-2.5[ | [1.5-2.5[ | **-> Z-heterosome** |
