# Supplementary material for: New WGS data and annotation of the heterosomal vs. autosomal localization of Ostrinia scapulalis (Lepidoptera, Crambidae) nuclear genomic scaffolds
Source: Data Brief. 2018 Aug 9;20:644–8. doi: 10.1016/j.dib.2018.08.011 (PMC6127984; doi:10.1016/j.dib.2018.08.011)
Supplement: Supplementary file 6 — Supplementary material [file mmc6.docx]

**Supplementary file 3. *A posteriori* validation of the AD-ratio method through blastn of known candidate genes against OSCA nuclear reference**

The different candidate genes were probably split across different scaffolds, as revealed by high alignment length and high similarity with multiple scaffolds. Most of the known gene were properly annotated, except *OR2*, which is known to be autosomal and which aligned against three scaffolds: one annotated as ‘autosomal’, one annotated as ‘putatively Z-heterosomal’ and one unannotated. Nevertheless, the best e-value was, in most cases, sufficient to validate scaffolds annotation.

Z-heterosomal scaffolds seemed to have been well annotated, even in cases of a lack of reproducibility between replicates, see *OR1*, *OR3* and *OR6*. Such *a posteriori* cross-validation reveals the power of the *AD-ratio* method to retrieve the autosomal or heterosomal localization of assembly fragments, such as scaffolds or contigs. However, ambiguous annotations should be taken with caution and we strongly recommend to discard markers located on ambiguously annotated scaffolds in further evolutionary genomics studies.

| **Candidate gene** | **Gene Accession (Genbank)** | **Scaffold** | **Scf**  **size** | **% id** | **Alignment length** | **E-value** | **Annotation AD-ratio** |
| --- | --- | --- | --- | --- | --- | --- | --- |
| OR1 | FJ385012.1 | newscf_6616 | 20830 | 96.67 | 480 | 0 | NA |
| (Z-heterosomal) |  | newscf_7058 | 12540 | 94.53 | 402 | 2.00E-176 | Heterosome |
|  |  | newscf_513 | 19138 | 100 | 158 | 2.00E-83 | Putative heterosome |
|  |  | newscf_1365 | 107041 | 86.07 | 122 | 2.00E-21 | Putative heterosome |
| OR2 | GQ844877.1 | newscf_7503 | 16415 | 99.08 | 218 | 2.00E-114 | Autosome |
| (autosomal) |  | newscf_998 | 44909 | 98.17 | 218 | 1.00E-109 | Putative heterosome |
|  |  | newscf_33824 | 1370 | 97.21 | 215 | 4.00E-103 | NA |
| OR3  (Z-heterosomal) | FJ385014.1 | newscf_1365 | 107041 | 99.19 | 493 | 0 | Putative heterosome |
|  |  | newscf_7058 | 12540 | 99.22 | 256 | 4.00E-137 | Heterosome |
|  |  | newscf_513 | 19138 | 86.07 | 122 | 2.00E-21 | Putative heterosome |
| OR4 | FJ385013.1 | newscf_2328 | 77594 | 97.1 | 482 | 0 | Autosome |
| (autosomal) |  | newscf_25926 | 1958 | 95.64 | 482 | 0 | Autosome |
| OR5 (autosomal) | FJ385015.1 | newscf_2328 | 77594 | 100 | 256 | 6.00E-142 | Autosome |
| OR6 | GQ844881.1 | newscf_8038 | 11441 | 98.78 | 490 | 0 | Heterosome |
| (Z-heterosomal) |  | newscf_16790 | 3844 | 99.54 | 217 | 3.00E-116 | Heterosome |
|  |  | newscf_1296 | 50891 | 100 | 158 | 2.00E-83 | Putative heterosome |
| KET  (Z-heterosomal) | EF396373.1 | newscf_106 | 41066 | 99.38 | 645 | 0 | Heterosome |
| TPi  (Z-heterosomal) | EF396447.1 | newscf_1555 | 232002 | 96.49 | 1565 | 0 | Heterosome |
